# Morphometric characterisation of wing feathers of the barn owl *Tyto alba pratincola *and the pigeon *Columba livia*

**DOI:** 10.1186/1742-9994-4-23

**Published:** 2007-11-21

**Authors:** Thomas Bachmann, Stephan Klän, Werner Baumgartner, Michael Klaas, Wolfgang Schröder, Hermann Wagner

**Affiliations:** 1 RWTH Aachen University, Institute of Biology II, Aachen, Germany; 2 RWTH Aachen University, Institute of Aerodynamics, Aachen, Germany

## Abstract

**Background:**

Owls are known for their silent flight. Even though there is some information available on the mechanisms that lead to a reduction of noise emission, neither the morphological basis, nor the biological mechanisms of the owl's silent flight are known. Therefore, we have initiated a systematic analysis of wing morphology in both a specialist, the barn owl, and a generalist, the pigeon. This report presents a comparison between the feathers of the barn owl and the pigeon and emphasise the specific characteristics of the owl's feathers on macroscopic and microscopic level. An understanding of the features and mechanisms underlying this silent flight might eventually be employed for aerodynamic purposes and lead to a new wing design in modern aircrafts.

**Results:**

A variety of different feathers (six remiges and six coverts), taken from several specimen in either species, were investigated. Quantitative analysis of digital images and scanning electron microscopy were used for a morphometric characterisation. Although both species have comparable body weights, barn owl feathers were in general larger than pigeon feathers. For both species, the depth and the area of the outer vanes of the remiges were typically smaller than those of the inner vanes. This difference was more pronounced in the barn owl than in the pigeon. Owl feathers also had lesser radiates, longer pennula, and were more translucent than pigeon feathers. The two species achieved smooth edges and regular surfaces of the vanes by different construction principles: while the angles of attachment to the rachis and the length of the barbs was nearly constant for the barn owl, these parameters varied in the pigeon. We also present a quantitative description of several characteristic features of barn owl feathers, e.g., the serrations at the leading edge of the wing, the fringes at the edges of each feather, and the velvet-like dorsal surface.

**Conclusion:**

The quantitative description of the feathers and the specific structures of owl feathers can be used as a model for the construction of a biomimetic airplane wing or, in general, as a source for noise-reducing applications on any surfaces subjected to flow fields.

## Background

Owls have evolved several specialisations for sound localisation: e.g. sound-reflecting feathers on the head [1], asymmetrically arranged ear flaps [2,3] and increased nuclei in the auditory pathway [4-6]. This is why the owl proved to be an excellent model system for studying prey capture in the last decades [2,4-7].

Its hunting strategy depends upon a low speed and silent flight in order to be able to locate the prey mainly by hearing and to avoid being heard early. For this reason, another specific of the owl is the fine structure of its feathers. The owl's feathers are equipped with special structures [8-10] that reduce noises of frequencies more than 2 kHz [11]. Thus, flight noise is reduced within the typical hearing spectrum of the owl's prey [11,12] and also within the owl's own best hearing range [13,14].

Pigeons, as other birds are commonly known for a noticeable noise production during flight, for instance a high frequency sound of rubbing feathers or noises generated by clap and fling [11,15].

A comprehensive qualitative investigation of morphological and functional aspects of bird feathers was provided by Sick [16]. However, he did not provide any quantitative morphometric data. In this paper, his biological terms for the fine structures of feathers will be used. The wing consists of imbricate feathers leading to a species-dependent wing planform, thickness distribution and camber line. These attributes influence the air flow over the wing. For the owl, the leading edge comb-like serrations, the trailing edge fringes on each feather and their velvet-like upper surface are additional parameters relevant for aerodynamics. Although Graham [8] described these structures to some extent, he neither presented detailed morphometric data nor did he give any explanations of the underlying mechanisms that lead to a reduction of noise.

Kroeger et al. [17] carried out several experiments in order to investigate the noise emission of free flying owls. They came to the conclusion that the noise emission of owls is different to other birds and also different to the airframe noise of airplanes and gliders. But neither did they study the morphometry of wings and flight feathers nor were they able to clarify the fundamental mechanism of noise reduction either.

Experiments with a focus on the leading edge and the comb-like serrations were carried out by Arndt and Nagel [18] and by Schwindt and Allen [19]. In their studies, Arndt and Nagel drew the conclusion that serrations function more as effective reducers of aerodynamic disturbances, than as a noise-reducer. Schwindt and Allen [19], however, concluded that serrations reduce air pressure on the leading edge.

Neuhaus et al. [11] compared the flight of the tawny owl (*Strix aluco*) and the mallard duck (*Anas platyrhynchos*). Their studies came up with significant differences in the morphology and the air flow of those two species. Specifically the investigation of the flow patterns revealed that the airflow around the primaries of the owl wing was laminar, while the duck wing showed large turbulent structures on the upper and lower side. After removing the comb-like serrations on the leading edge of the owl's wing, the laminar flow changed to turbulent resulting in a separation closer to the leading edge instead of reducing any noise.

Even though there is some information available on the mechanisms that lead to a reduction of noise emission, neither the morphological basis, nor the biological mechanisms of the owl's silent flight are known. Therefore, a systematic program to study the morphology of the barn owl's (*Tyto alba*) wing, and to compare it to a non-specialist, the pigeon (*Columba livia*), was initiated.

This report presents a comparison between the feathers of the barn owl and the pigeon. The specific characteristics of the owl's feathers will be shown on macroscopic and microscopic level.

## Results

**Figure 1:**
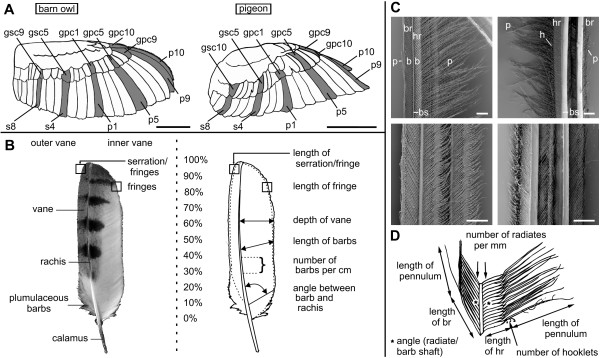
**Feather position and measured feather parameters**. (A) Position of the investigated feathers in the barn owl (left) and the pigeon (right); scale bar: 10 cm. (B) Investigated parameters on the flight feather (p5 of a barn owl). Measurements were taken every 10% of feather length. (C) Scanning electron microscopy pictures of two connected barbs at 60% of the inner vane of feather p10 of the barn owl (above) and the pigeon (below) from dorsal (left) and ventral (right) view (bs: barb shaft, hr: hook radiate, br: bow radiate, p: pennulum, h: hooklet); scale bar 200 μm. (D) Investigated barb parameters.

The wing feathers can be divided into remiges (or flight feathers) and coverts. Furthermore, both types of feathers can be subdivided into primaries and secondaries. This nomenclature is commonly used [20] and will be used here to describe the position of the feathers on the wing (Fig. 1A).

Five feathers p10 (Fig. 1A; for this and the further abbreviations please see List of abbreviations) from five wings of three different owls were examined; feathers from the other positions were taken from two wings of two individuals. The eight feathers p10 of the pigeon were taken from eight wings of four individuals; all other feathers from two wings of two individuals. Fig. 1A shows the positions of the examined feathers. In the following, the relevant morphological characteristics of the feathers (Fig. 1B) as outlined in the Methods section will be presented. Afterwards, measurements of the important parameters of the barbs are presented (see Fig. 1C,D). The data on the feathers of the owl will be compared to the corresponding data on the pigeon feathers.

### Characteristics of feathers

The feather consists of a shaft (rachis) and a vane. The vane is divided into an outer and inner part by the rachis (Fig. 1B). The basal part of the rachis, called quill, is embedded in the bird's skin and therefore does not show any barbs. The examination included the depth, size and shape of the inner and outer vanes. The results of the measurements of the feathers p10 varied only marginally (SEM of approximately 6% for the barn owl and less than 3% for the pigeon) (Table 1). Therefore, only two feathers of the other positions were examined.

**Table 1:** Morphometric parameters of barn owl's and pigeon's remiges

barn owl		p10	p9	p5	p1	s4	s8
length of rachis	[cm]	24.49^a ^+/- 0.58	27.40	21.86; 22.50	17.36	15.41; 17.64	16.55; 15.96
area of outer vane	[cm^2^]	10.44^a ^+/- 0.65	19.56; 20.11	18.59; 21.15	12.82; 16.78	14.95; 18.82	16.11; 15.85
area of inner vane	[cm^2^]	52.15^a ^+/- 3.31	62.60; 65.93	48.15; 55.90	30.79; 38.44	34.73; 40.48	35.35; 38.57
AI (area)		-0.67	-0.53	-0.45	-0.4	-0.38	-0.4
l_serr_^a^/l_fr_^b ^ov	[mm]	1.8^c ^+/- 0.06	2.03^d ^+/- 0.22	3.62^d ^+/- 0.4	4.33^d ^+/- 0.68	3.29^d ^+/- 0.45	3.21^d ^+/- 0.51
l_fr_^b ^inner vane	[mm]	3.45^d ^+/- 0.15	3.21^d ^+/- 0.18	1.82^d ^+/- 0.16	1.68^d ^+/-0.2	1.88^d ^+/- 0.28	1.97^d ^+/- 0.3
							

pigeon		p10	p9	p5	p1	s4	s8

length of rachis	[cm]	19.31^b ^+/- 0.21	19.63	16.71	11.62	11.35	10.33
area of outer vane	[cm^2^]	4.33^b ^+/- 0.11	7.07; 6.67	8.09; 8.69	8.18; 7.82	10.14; 11.72	9.70; 9.62
area of inner vane	[cm^2^]	17.58^b ^+/- 0.40	19.00; 21.50	17.10; 20.03	11.18; 11.70	11.52; 12.84	9.89; 10.94
AI (area)		-0.6	-0.5	-0.38	-0.18	-0.05	-0.04

^a ^mean values with standard error of the mean, N = 5

^b ^mean values with standard error of the mean, N = 8

^c ^mean length of serration with standard error of the mean, N = 16 (p10: N = 40)

^d ^mean length of fringe with standard error of the mean, N = 16 (p10: N = 40)

**Figure 2:**
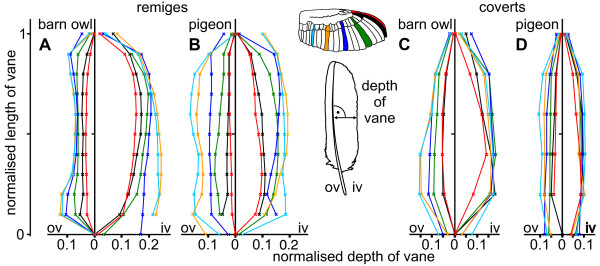
**Depth of vane in barn owl and pigeon wing feathers**. Normalised depth of the outer and inner vane of remiges (A, B) and coverts (C, D) in the barn owl (A, C) and the pigeon (B, D). The colours indicate different feathers. Their position is presented at the wing. The depth was measured at right angles to the rachis and was then normalised with respect to the whole length of vane.

Barn owls and pigeons were nearly of the same body weight, but owl feathers were typically larger than pigeon feathers, resulting in a larger wing span, wing area and wing chord. For example, the feather p10 of the barn owl had a mean length of 24.5 cm, while the length of the pigeon's feather p10 was only 19.3 cm (Table 1). For both species, feather p10 was the second longest and was only exceeded by feather p9. This difference in size could also be found in the vane's depth. The normalised depth of the inner and outer vanes varied (Fig. 2) with the depth of the barn owl's outer vanes of the primaries being larger than that of the pigeon. Especially for the feather p10 the difference between the two species was obvious. The comparison of the outer vanes of the secondaries showed a contrary result: The outer vanes of the pigeon's secondaries were larger than those of the barn owl (compare Fig. 2A with Fig. 2B).

**Figure 3:**
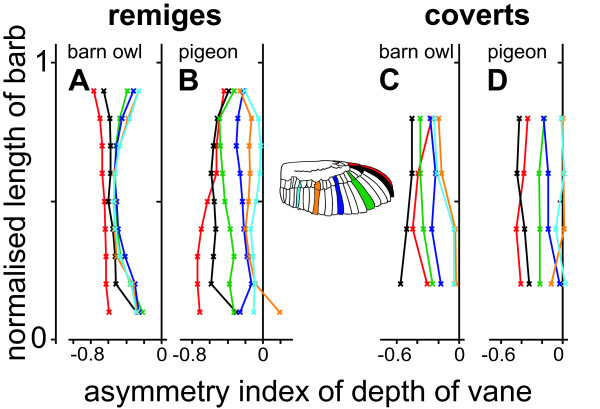
**Asymmetry of barn owl and pigeon wing feathers**. Asymmetry index of depth. Presents the asymmetry of the depth in the remiges (A, B) and coverts (C, D) in the barn owl (A, C) and the pigeon (B, D). The colours indicate different feathers. Their position is presented at the wing.

The depth of the vane as a function of its length was measured and the mean value was calculated. For all examined feathers (except for the secondary coverts in the pigeon), the outer vane was smaller than the inner vane (Fig. 2). The asymmetry index AI_d_, introduced in the Methods section (Eqn. 1), revealed two morphometric characteristics (Fig. 3): On the one hand, the asymmetry in the pigeon's remige was smaller and, on the other hand, showed a much higher variation along the length of the feather compared to the barn owl. The mean asymmetry of the pigeon's remiges decreased from lateral to medial (p10, AI_d _= -0.61; p1 AI_d _= -0.23; s8, AI_d _= -0.1), whereas the asymmetry of the barn owl's remiges changed only little (p10, AI_d _= -0.66; p1, AI_d _= -0.44; s8, AI_d _= -0.42) (for position of the feathers see Fig. 1A).

The distribution of the coverts' asymmetry in the barn owl and in the pigeon was similar to the distribution of the pigeon's remiges (Fig. 3C,D). Only the greater secondary coverts of both species differed. A clear asymmetry could be found in the barn owl's coverts, in contrast to the pigeon's coverts, where the secondary coverts were almost symmetrical. However, the asymmetry of the coverts' vanes was typically smaller than the asymmetry of the primaries (Fig. 3C,D).

The feathers of both species showed different shapes. This can be seen in the normalised depth of vanes. For example feather p10 of the barn owl had its maximum of depth of the inner vane at approximately 70% of the vane's length (Fig. 2A). For the pigeon, the maximum of depth of feather p10 was located at 40% of the vane's length (Fig. 2B). The inner vane of the pigeon's feather p10 had a characteristic emargination at 60% of its length in all eight investigated feathers (Fig. 2B). This emargination was unique in feather p10 and could not be found in any other feather.

Only marginal changes in depth were observed in the outer vanes of the feathers p10 and p9 ranging at 10% to 90% of its length (Fig. 2A,B). This observation applied for both species. The outer vanes of the other remiges formed a double s-shape curve with two bulges in the first and last quarter. This shape was more pronounced in the barn owl's feathers (Fig. 2A,B). The bulge at the tip of the feather was mainly due to curvature of the rachis and not due to changes in the feather's margin. The bulge at the base of the feather was mainly due to the plumulaceous barbs. The combination of the s-shape of the outer vane and the ellipsoid shape of the inner vane resulted in a maximum of AI_d _at 50% of the normalised length for most remiges of the barn owl (Fig. 3A).

The coverts of the barn owl showed noticeable changes along the vane's length. These changes occurred in the first 40% and were most obvious on the outer vane for gsc5 and gsc9 (Fig. 2C). In the first 40%, these feathers lack a closed vane because of the plumulaceous barbs. Pigeon coverts also had plumulaceous barbs, but they did not result in a larger depth at the feather's base. The pigeon coverts were slim and elongated in the normalised depiction (normalised depth of vane below 0.12), while the barn owl's coverts were wider (normalised depth of vane up to 0.2) (Fig. 2C,D).

The area of the outer and inner vanes was measured and an asymmetry index (AI_a_, Eqn. 2, see Methods section) was calculated as described in the Methods section. For example, the outer vane of the feather p10 of the pigeon had an area of 4.33 cm^2^, while the inner vane had an area of 17.58 cm^2^, resulting in an asymmetry index of -0.6 (Table 1). With a mean area of 10.44 cm^2 ^for the outer vane of feather p10 and 52.15 cm^2 ^for the inner vane, resulting in an asymmetry index of -0.67, the barn owl's vane was approximately three times larger than the pigeon's. The asymmetry of the feather depended upon its position on the wing. The highest asymmetry could be found in the feathers p10, the lowest in the feathers s4 and s8. The absolute values of AI_a _differed between pigeon and barn owl (Table 1, 2). For the barn owl, the asymmetry was higher, but the tendency of more asymmetric primaries was the same for both species.

**Table 2:** Morphometric parameters of barn owl's and pigeon's coverts

barn owl		gpc10	gpc9	gpc5	gpc1	gsc5	gsc9
length of rachis	[cm]	5.74; 6.27	8.55; 8.97	9.83; 10.30	8.47; 8.96	8.14; 9.10	7.65
area of outer vane	[cm^2^]	1.80; 1.85	3.43; 3.41	7.64; 8.52	7.95; 8.22	11.28; 12.11	9.05; 9.20
area of inner vane	[cm^2^]	3.09; 3.55	7.45; 8.71	12.47; 14.89	10.73; 11.34	12.62; 13.93	10.92; 12.42
AI (area)		-0.29	-0.41	-0.26	-0.15	-0.06	-0.12
l_serr_^a^/l_fr_^b ^ov	[mm]	1.46^a ^+/- 0.16	1.91^b ^+/- 0.51	1.53^b ^+/- 0.54	3.46^b ^+/- 0.33	7.18^b ^+/- 2.9	5.89^b ^+/- 2.58
l_fr_^b ^inner vane	[mm]	6.1^b ^+/- 1.57	4.63^b ^+/- 0.69	5.66^b ^+/- 1.22	3.78^b ^+/- 0.71	4.74^b ^+/- 1.02	4.44^b ^+/- 1.26
							

pigeon		gpc10	gpc9	gpc5	gpc1	gsc5	gsc10

length of rachis	[cm]	4.32; 4.59	6.88	8.36	6.91	7.18; 7.75	6.24; 7.26
area of outer vane	[cm^2^]	0.65; 0.65	1.53; 1.93	3.47; 3.72	5.36; 3.43	6.35; 6.84	5.63; 7.44
area of inner vane	[cm^2^]	1.41; 1.19	3.84; 3.62	4.96; 5.70	6.12; 4.17	5.92; 6.65	5.04; 6.56
AI (area)		-0.33	-0.37	-0.19	-0.08	0.02	0.06

^a ^length of serration with standard error of the mean, N = 8

^b ^mean length of fringe with standard error of the mean, N = 8

### Characteristics of the barbs

The vane consists of barbs. Therefore, a closer investigation of the barbs revealed the fine structure of a feather. The parameters introduced in the Methods section (Fig. 1C,D) were determined and a comparison between the barn owl and the pigeon was made in order to point out the special structures which evolved in the owl. At first, a general comparison will be given. Following, data on the special structures of the owl's feathers, which are the serrations, the fringes and the velvet-like surface, will be presented.

**Figure 4:**
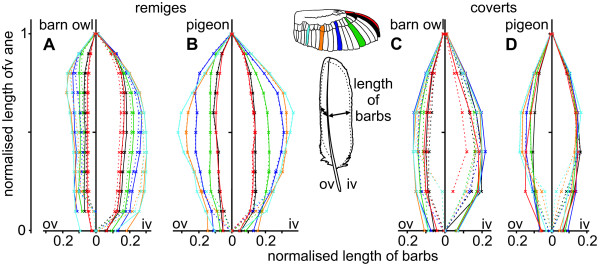
**Length of barbs in barn owl and pigeon wing feathers**. The normalised length of barbs of the inner (iv) and outer vane (ov) of remiges (A, B) and coverts (C, D) from the barn owl (A, C) and the pigeon (B, D) are shown. The length was measured from the base to the tip and then normalised with respect to the whole length of vane. The area outside the dotted lines indicates regions of unconnected barbs forming the plumulaceous barbs (in both species), the fringes (in the barn owl) or serrations (p10 and gpc10 in the barn owl).

**Figure 5:**
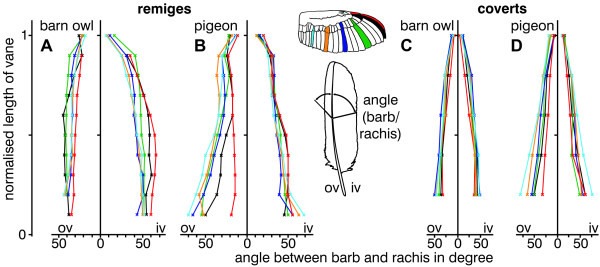
**Angle between barbs and rachis of wing feathers of the barn owl and the pigeon**. Demonstrates the angles between the barbs and the rachis at the inner (iv) and outer vane (ov) of remiges (A, B) and coverts (C, D) from the barn owl (A, C) and the pigeon (B, D). The colours indicate different feathers. Their position is presented at the wing.

In both species, the outer vane was homogeneous, which means that the edge was smooth and the surface regular. However, by taking a closer look at the length of the barbs and the angle of attachment of the barbs to the rachis, two different principles of construction were revealed. The length (Fig. 4A) and the angle of attachment (Fig. 5A) of the barn owl's barbs were nearly constant. By contrast, the pigeon's barbs varied in both parameters (Fig. 4B, Fig. 5B). The normalised length of the barbs increased towards the middle of the rachis (feather centre) and decreased towards the tip (Fig. 4B). The highest increase was found at the pigeon's feather s8. Here, the length of the barbs increased by a factor of three compared to those at the base of the feather. For most feathers of the pigeon, the angle of attachment decreased from the base to the tip of the feather. The change in the angle of attachment together with the variation of the length resulted in an almost constant depth of the outer vane as can be seen in Fig. 2B. The pigeon's feather p10 and all feathers of the barn owl had an almost constant angle of attachment. The angle of attachment of the barbs of the inner vane showed a similar, but less pronounced distribution than that of the outer vane. The most acute as well as the most obtuse angle was measured at the inner, respectively at the outer vane of feather p10 for both species.

The barbs of the coverts did not reveal interspecific differences in length as big as was observed in the primaries (Fig. 4). The normalised length of the barbs of the barn owl's coverts increased slightly to the centre of the vane (Fig. 4C). The angle of attachment of the inner and the outer vanes decreased in an almost linear way in the owl and the pigeon. However, the differences between feathers from different positions were smaller in the barn owl (compare Fig. 5C with Fig. 5D).

**Figure 6:**
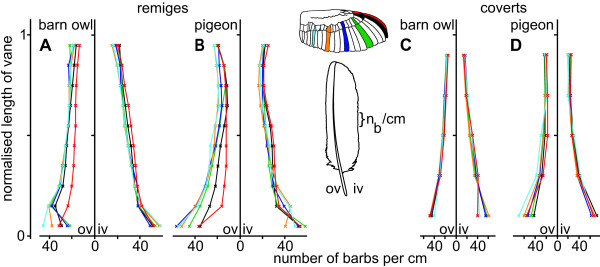
**Barb density of wing feathers of the barn owl and the pigeon**. The number of barbs per cm at the inner (iv) and outer vane (ov) of remiges (A, B) and coverts (C, D) from the barn owl (A, C) and the pigeon (C, D) are shown. The mean barb density per cm was calculated by dividing the total number of barbs by 10% (respectively 20%) of vane's length. The colours indicate different feathers. Their position is presented at the wing.

The area outside the dotted lines in Fig. 4 represents regions of unconnected barbs. They play a decisive role in the description of barbs, because they form the plumulaceous barbs and the fringes of the feather edges. In one special case, they also form the serrations on the outer vane of the feathers p10 and gpc10 of the barn owl. This special structure will be discussed later. By and large, the density of the barbs was independent from the species (Fig. 6). Moreover, the variation of the density, depending on the position of the feather, was smaller than the variations measured in length and angle (compare Fig. 6 with Fig. 4 and 5). The largest variation occurred in the area of the plumulaceous barbs. This was typically in the range from 0 to 0.1 of the normalised length of the feather. The outer vanes of the pigeon's remiges showed the greatest variation along their length as well as compared to feathers from other positions (Fig. 6B).

The number of barbs of the remiges decreased towards the tip of the vane. In contrast to the barn owl, the number of barbs of the pigeon's remiges increased slightly at the tip of the vane. Interestingly, the outermost feathers (p10; gpc10) had the lowest density of barbs on the outer vane, but a very high density on the inner vane. This was observed for both species. Again, the interspecific differences in barb density were less distinct in the coverts than in the remiges.

**Figure 7:**
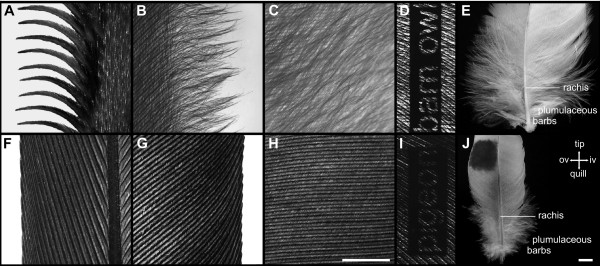
**Details of a feather**. (A-E) Details of the barn owl's feather p10. (A) Serrations at the outer vane's leading edge. (B) Fringes at the inner vane's trailing edge. (C) Velvet-like dorsal surface of the inner vane. (F-J) Details of the pigeon's feather p10. (F) Leading edge of the outer vane. (G) Trailing edge of the inner vane. (H) Dorsal surface of the inner vane; scale bar: 1 mm. (D, I) Qualitative illustration of the porosity (translucency) of black dyed inner vanes of feather gsc5 of the barn owl (D) and the pigeon (I). (E, J) Plumulaceous barbs of feather gsc5 of the barn owl (E) and the pigeon (J); scale bar: A-D and F-I: 1 mm, E and J: 5 mm.

The leading edge of the barn owl's feathers p10 and gpc10 formed comb-like serrations (Fig. 7A). These structures could not be found in any other feather. Each serration was formed by the tip of a single barb and might be divided into a proximal base and a distal, tooth-shaped tip (Fig. 7A). The shape of each serration was curved in a way that the tip was pointing towards the proximal end of the feather (Fig. 7A). Additionally, each serration was bent to the dorsal side. The tooth-shaped tip had a mean length of 1.8 mm (Table 1). The mean density of serrations was 18/cm, which was, naturally, equivalent to the barb density as shown in Fig. 6A (red line, outer vane). Therefore, the base of each serration was 555 μm wide. The width of the serrations tapered in an almost linear mode towards the tip, resulting in a mean width of 254 μm (+/- 4.3 SEM) at 50% of its length.

Barbs do not only build the vane, but also the edges of the feathers and thus the edges of the wing. The barn owl evolved fringes at the edges of their feathers (Fig. 7B). A fringe is formed by the tip of a barb. In the region of fringes, hook and bow radiates were present. However, they were not connected, because the hook radiates lacked hooklets. Additionally, the barb shafts became thinner towards their ends. Therefore, the barb ends could float freely (Fig. 7B). Fringes were found on the outer as well as on the inner vane of almost each investigated barn owl remige and covert. The only exceptions were the outer vanes of feathers p10 and gpc10 of the barn owl, because the barbs formed serrations. Fringes on the inner vanes were more obvious than those of the outer ones. The fringes on the outer vanes were shorter and often oriented parallel to the leading edge of a feather. A typical fringe on the inner vane of the barn owl's feather p10 had a mean length of 3.45 mm (+/-0.15 SEM) (Table 1). The fringes on the inner vane were smallest in feather p1 with a mean length of 1.68 mm (+/-0.2 SEM) (Table 1). The mean calculated density of the barbs on the inner vanes of the barn owls' remiges was 28.5/cm (+/- 0.76 SEM) resulting in a spacing of 358 μm between two fringes. The edges of the pigeon's feathers were typically smooth or slightly undulated (Fig. 7D,E). The hooklets of the radiates at the end of the barbs remained connected and therefore did not form any fringes. The only area in which unconnected barbs were found was the region of plumulaceous barbs at the base of the inner and outer vanes (Fig. 7I). Only in this area fringe-like structures were formed. In the coverts of the barn owl, the fringes were even more distinct than in the remiges (between 3.78 mm and 6.1 mm). Once again, the coverts of the pigeon did not have fringes apart from the plumulaceous barbs.

### Characteristics of the radiates

Hook radiates (distal barbules) are distal extensions from the barbs, while bow radiates (proximal barbules) are proximal extensions. Each radiate can be divided into a base and a pennulum [16]. Radiates did not show many intraspecific differences, not even between remiges and coverts. However, interspecific differences occurred (Fig. 1C). The average mean density of hook radiates over all investigated feathers was determined to 31 (+/-5) per mm for the barn owl (Table 3). The pigeon's averaged mean density was higher (44 (+/-6) per mm). The number of bow radiates (br) was lower than the number of hook radiates (hr) for all investigated feathers of both species (barn owl br/hr = 0.71; pigeon br/hr = 0.73, Table 3). We observed barn owl feathers to be more porous than pigeon feathers which could also be seen in different translucency. This higher porosity in the barn owl is a consequence of the lesser density of radiates than in the pigeon. To demonstrate this qualitatively, we dyed vanes of barn owl and pigeon feathers with black hair tinting lotion to avoid influences of different keratin colours. Afterwards, a transparent foil with writing (barn owl, pigeon) was placed between feathers and light source. The feathers were illuminated from below and each feather was photographed using the same resolution and exposure time. In contrast to the pigeon, the barn owl lettering could easily be recognised (Fig. 7D,I). A quantitative description of the different porosity in both species can be found in Table 3 (number of barbules).

**Table 3:** Parameters of barbs of barn owl's and pigeon's wing feathers

		barn owl					pigeon				
	lb_n_^2^	n_hr_^1^	n_br_^1^	n_h_^1^	α_hr_^1^	α_br_^1^	n_hr_^1^	n_br_^1^	n_h_^1^	α_hr_^1^	α_br_^1^

p10	0.75	35 +/- 1.1	28 +/- 0.6	1 +/- 0.3			50 +/- 2.4	39 +/- 1.3	6 +/- 0.2	23 +/- 1.4	21 +/- 1.3
outer	0.5	39 +/- 0.7	30 +/- 0.5	3 +/- 0.1	50 +/- 1.3	36 +/- 1.6	51 +/- 2.0	36 +/- 0.8	6 +/- 0.1	32 +/- 1.9	27 +/- 1.3
vane	0.25	42 +/- 0.8	32 +/- 0.9	3 +/- 0.1	56 +/- 1.1	38 +/- 1.8	55 +/- 1.6	37 +/- 0.7	5 +/- 0.2	44 +/- 1.2	34 +/- 1.0
											
p10	0.8	26 +/- 0.8	18 +/- 0.6	4 +/- 0.1	40 +/- 1.4	16 +/- 0.9	43 +/- 1.1	31 +/- 0.8	5 +/- 0.4	33 +/- 2.6	21 +/- 1.0
inner	0.6	27 +/- 0.6	18 +/- 0.3	4 +/- 0.1	41 +/- 1.2	17 +/- 0.9	48 +/- 0.7	48 +/- 0.7	4 +/- 0.3	42 +/- 1.4	25 +/- 0.8
vane	0.4	31 +/- 0.6	18 +/- 0.4	3 +/- 0.1	48 +/- 1.6	21 +/- 1.0	55 +/- 1.9	33 +/- 0.5	3 +/- 0.1	49 +/- 1.6	28 +/- 0.7
	0.2	36 +/- 0.7	19 +/- 0.5	3 +/- 0.1	60 +/- 1.6	24 +/- 1.7	56 +/- 1.9	34 +/- 0.8	4 +/- 0.1	49 +/- 1.4	33 +/- 0.9
											
s8	0.8	26 +/- 1.3	22 +/- 1.5	2 +/- 0.5	29 +/- 3.0	25 +/- 2.0	42 +/- 1.9	32 +/- 1.8	4 +/- 0.6	34 +/- 1.4	29 +/- 0.6
outer	0.6	27 +/- 0.9	21 +/- 0.8	3 +/- 0.2	39 +/- 1.2	25 +/- 0.7	39 +/- 0.9	30 +/- 1.1	5 +/- 0.3	34 +/- 0.6	27 +/- 0.6
vane	0.4	30 +/- 0.3	22 +/- 0.4	3 +/- 0.1	43 +/- 1.3	30 +/- 1.4	41 +/- 0.8	30 +/- 0.4	5 +/- 0.2	38 +/- 0.5	22 +/- 1.0
	0.2	35 +/- 0.8	24 +/- 0.8	3 +/- 0.1	51 +/- 1.1	31 +/- 0.5	41 +/- 1.0	30 +/- 0.5	5 +/- 0.2	42 +/- 1.2	20 +/- 1.5
											
s8	0.8	34 +/- 1.2	21 +/- 0.7	3 +/- 0.1	44 +/- 1.1	19 +/- 0.8	39 +/- 1.7	30 +/- 1.4	5 +/- 0.2	41 +/- 1.5	25 +/- 0.8
inner	0.6	32 +/- 1.1	19 +/- 0.4	3 +/- 0.1	45 +/- 1.1	20 +/- 0.8	37 +/- 0.8	28 +/- 0.8	5 +/- 0.1	44 +/- 1.4	26 +/- 0.8
vane	0.4	31 +/- 0.6	18 +/- 0.2	3 +/- 0.1	49 +/- 1.0	17 +/- 0.5	41 +/- 1.0	30 +/- 0.9	5 +/- .01	39 +/- 0.8	20 +/- 0.7
	0.2	34 +/- 0.7	21 +/- 0.5	3 +/- 0.1	58 +/- 0.8	24 +/- 0.8	40 +/- 1.2	31 +/- 0.5	5 +/- 0.1	42 +/- 1.5	21 +/- 2.0
											
gpc1	0.8	23 +/- 0.4	20 +/- 0.8	2 +/- 0.2	35 +/- 2.6	29 +/- 2.3	39 +/- 1.4	30 +/- 1.2	5 +/- 0.2	22 +/- 1.3	16 +/- 0.4
outer	0.6	25 +/- 0.4	21 +/- 0.6	3 +/- 0.1	24 +/- 4.3	15 +/- 3.1	42 +/- 1.7	30 +/- 1.0	4 +/- 0.2	27 +/- 1.3	21 +/- 0.6
vane	0.4	30 +/- 0.7	22 +/- 0.9	3 +/- 0.2	42 +/- 0.7	28 +/- 0.5	44 +/- 1.3	31 +/- 1.0	4 +/- 0.1	36 +/- 0.8	26 +/- 0.7
	0.2	33 +/- 1.0	23 +/- 0.5	3 +/- 0.1	45 +/- 0.8	39 +/- 1.9	42 +/- 1.3	31 +/- 1.0	4 +/- 0.2	43 +/- 1.4	29 +/- 0.7
											
gpc1	0.8	27 +/- 3.5	24 +/- 3.0	2 +/- 0.4	32 +/- 1.3	25 +/- 0.7	38 +/- 1.2	29 +/- 1.5	5 +/- 0.6	35 +/- 1.2	16 +/- 0.3
inner	0.6	28 +/- 2.0	22 +/- 1.8	3 +/- 0.1	25 +/- 4.4	26 +/- 0.2	38 +/- 0.7	27 +/- 1.2	4 +/- 0.1	36 +/- 1.0	19 +/- 0.4
vane	0.4	31 +/- 1.5	22 +/- 1.6	3 +/- 0.1	42 +/- 1.3	17 +/- 3.1	40 +/- 1.0	28 +/- 0.7	4 +/- 0.1	42 +/- 0.9	23 +/- 0.5
	0.2	34 +/- 1.0	23 +/- 0.9	3 +/- 0.2	45 +/- 1.2	18 +/- 0.5	42 +/- 1.6	30 +/- 0.9	4 +/- 0.2	49 +/- 1.6	29 +/- 0.5

^1 ^mean values with standard error of the mean, N = 16

^2 ^normalised length of barb; 1 = tip of barb

The pigeon's hook radiates had typically more hooklets (5) than those of the barn owl (3) (Table 3). The number of hooklets decreased towards the tip of the feather (not shown) on both, the inner and outer vane. For both species, the hook radiates were always attached in a more acute angle than the bow radiates (Table 3, Fig. 1C). The largest angles were found in the barn owl and varied between 24 and 60 degrees (Table 3). The pigeon's hook radiates were attached in an angle of 22 to 49 degrees (Table 3). The interspecific difference in the angle between bow radiates and barb shaft was smaller (barn owl: 15–39 degrees, pigeon: 16–34 degrees) (Table 3). No clear differences could be found between remiges and coverts.

**Figure 8:**
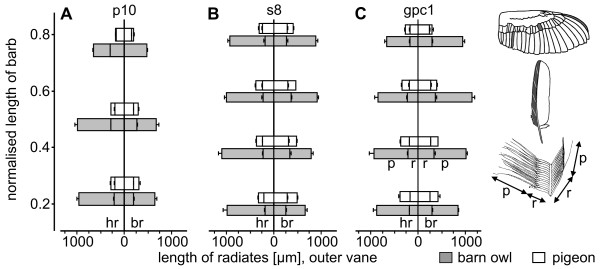
**Length of radiates of the outer vane of three different wing feathers from the barn owl and the pigeon**. The mean length of the radiates of the outer vanes of the feathers p10, s8 and gpc1 in the barn owl (grey) and the pigeon (white) is depicted. Each diagram is divided into hook radiates (hr-left) and bow radiates (br-right). Additionally, each radiate was divided into radiate base (r) and pennulum (p). SEM: standard error of the mean.

**Figure 9:**
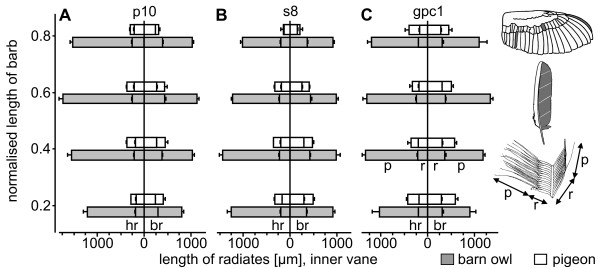
**Length of radiates of the inner vane of three different wing feathers from the barn owl and the pigeon**. The mean length of the radiates of the inner vanes of the feathers p10, s8 and gpc1 in the barn owl (grey) and the pigeon (white). Each diagram is divided into hook radiates (left) and bow radiates (right). Additionally, each radiate can be divided into radiate base and pennulum. SEM: standard error of the mean.

Length and shape of the radiates changed in the region of the serrations (Fig. 8A). They shortened towards the tip of the barb (Table 3) and the number of hooklets decreased to zero. The base of the bow radiates merged directly into the pennulum without a clear differentiation between both. Therefore, in Fig. 9A the total length of the bow radiates at 75% barb length is listed. The separation of barbs is mainly due to the lack of hooklets, shorter radiates and a change of the barb shaft in its form and shape. One serration tapered towards the tip and was bent in two different directions. As seen in Fig. 7A, the barb shaft was bent towards the feather base (calamus) and also to the dorsal side (not shown). Apart from the outer vanes of the feathers p10 and gpc1 (see above), every inner and outer vane of the barn owl was equipped with fringes. In the area of the fringes, the hooklets on the hook radiates were missing as well. By contrast to the serration, the bow and the hook radiates were not shortened. Thus, the fringes consisted of the unconnected elongated radiates and the barb shafts, leading to a fluffy structure (Fig. 7B).

The velvet-like appearance of the barn owl feathers was predominantly a consequence of elongated pennula (Fig. 1C, Fig. 7C). The pennula covered the barb shafts. This clearly differentiated the velvet-like structure of the barn owl's feathers from the homologue area in the pigeon (Fig. 7H), where a straight alignment of the barb shafts was obvious. A velvet-like structure could not be found on any of the pigeon's feathers. The length of the pennula was measured for feathers p10, s8 and gpc1 of both species on the outer vane as well as on the inner vane (Table 4). The mean length of the pennula of the inner and outer vane was larger in the barn owl than in the pigeon (Fig. 8, Fig. 9; Table 4). The pennula of the outer vane were always shorter than those of the inner vane (Fig. 8, Fig. 9; Table 3). For instance, an average pennulum of the barn owl's outer vane was 601 μm, while the average pennulum of the pigeon was 79 μm (Table 4). There was a greater divergence in the length of the inner vane, with the pigeons' pennulum length being 136 μm and the barn owls' pennulum being 1271 μm (Table 3). The pigeon's pennula were short and did not extend to the shaft of the next barb (Fig. 1C). By contrast, the barn owl's pennula overlapped up to four neighbouring barbs shafts (Fig. 1C). This difference in length was due to the length of the pennula and not to the length of the base of the radiates (Fig. 8, Fig. 9). We found that the length of pennula augmented in length in areas which were covered by another feather (Fig. 9). Remiges and coverts were arranged in an imbricate way meaning outer vanes overlapped wide ranges of inner vanes of adjacent feathers.

**Table 4:** Parameters of pennula (hook radiates)

Tyto alba	p10		s8		gpc1	
	outer vane	inner vane	outer vane	inner vane	outer vane	inner vane

density of pennula/mm^2 ^^a^	73.5	99.8	82.3	100.2	79.1	91.5
mean length of pennula [μm] ^b^	601 +/- 57.5	1271 +/- 87.1	785 +/- 43.3	1017 +/- 55.8	799 +/- 67.3	1014 +/- 63.4
						

Columba livia	p10		s8		gpc1	

	outer vane	inner vane	outer vane	inner vane	outer vane	inner vane

density of pennula/mm^2 ^^a^	84.8	152.5	114.1	109.5	154.5	135.1
mean length of pennula [μm] ^b^	79 +/- 6.6	136 +/- 15.0	171 +/- 7.8	181 +/- 10.7	158 +/- 14.1	201 +/- 22.3

^a ^mean values, n_b _(p10: N = 50, s8, gpc1: N = 20) multiplied with n_hr _(p10 ov: N = 48; all other N = 64)

^b ^mean values with standard error of the mean, N = 16

In both species the pennula of the outer vane of feather p10 decreased in length towards the tip (Fig. 8A). The radiates' length of the inner vane of the pigeon's 10^th ^primary remained nearly constant (Fig. 9A). By contrast, the barn owl's radiates of the inner vane of the 10^th ^primary increased in length towards the tip, especially the hook radiates (Fig. 8A, Fig. 9A). A similar effect could be noticed for all other investigated feathers of the barn owl. For instance, the feather gpc1 was positioned at the wrist (Fig. 1A). Hence, the main covered areas of this feather were found at its base (covered mainly by feathers of the median coverts (Fig. 1A)) and its inner vane (covered by feather gsc1 (Fig. 1A)). The pennula on the inner vane (Fig. 9C) were longer than those on the outer vane (Fig. 8C). From the density of barbs (e.g. 33.29/cm on the inner vane of p10) and the density of hook radiates (29.98/mm on the inner vane of p10) in the barn owl, we calculated the average density of the pennula to 99.8/mm^2 ^(Table 4). The average density for the homologue structure of the pigeon was 152.5/mm^2 ^(Table 4). By comparing the homologous structures of both species (Table 4) it was found that the density of pennula was higher for the pigeon than for the barn owl.

## Discussion

Data on the structure of feathers of the barn owl and pigeon was presented. Special attention was paid to the leading edge feather p10. In addition to that, data from 11 other positions, both from remiges and coverts, were included in this study. Apart from a general description, we specifically concentrated on the specialisations of the barn owl and compared them to the homologue structures of the pigeon. In the following, the acquired data will be discussed in respect to the data from other feathers and feathers from other species. Finally, the effects of these results on noise reduction will be discussed.

### Morphology of feathers

Pigeons and barn owls have approximately the same number of flight feathers, but those of the barn owl were larger. The wing area of the barn owl was much larger than that of the pigeon. Being approximately of the same weight, the wing area loading of the barn owl is lower, allowing it to fly very slowly. Neuhaus et al. [11] drew the same conclusion when comparing the tawny owl and the mallard duck.

Moreover, the asymmetry in size of the outer and inner vanes was more distinct for the barn owl than for the pigeon, especially for the remiges. An asymmetry is often found in remiges of flying birds [20] and is due to the functionality of bird flight [21]. Already Lilienthal [22] attributed different functions to the inner and outer vane in flapping flight. On the one hand, during the downstroke, the inner vanes are pressed towards the stiff raches and the outer vanes of the superimposed feathers, creating a closed airfoil. On the other hand, during the upstroke, the feathers separate, allowing the air to flow between the remiges through the wing [22]. Ennos et al. [23] developed a geometric model of the vane, specifically emphasising the importance of the interlinking of the barbs. This model suggests that the vane is more resistant to forces from below than from above, supporting the observations of Lilienthal [22]. The inner vanes of the barn owl's feathers were soft and pliant, due to the fact that they have fewer hook and bow radiates per mm and fewer hooklets per hook radiate. By contrast, the pigeon's vanes appeared to be stiffer. The vanes of the pigeon's feathers were connected more tightly because more radiates and more hooks per hook radiate were able to interlink. So, in case of the pigeon, the probability of a mechanical linkage between radiates and hooklets is obviously higher. Barn owl feathers were also more porous, which could be seen qualitatively by the translucency. This was due to the fact that barn owl feathers had a lesser density of barbules, but the same density of barbs, resulting in a more porous and translucent structure. As a consequence, the air may not only flow between the remiges, but also flow through a single feather from ventral to dorsal and vice versa. Interestingly, Mueller and Patone [24] measured the air flow through the vane. They found that inner vanes were less permeable for air than outer vanes. These authors proposed that the functional significance of this difference lies in the formation of a smooth, continuous wing surface. We suggest that a similar mechanism may underlie the construction of a large, closed and smooth wing surface also with the barn owl's soft and pliant feathers.

### Morphology of barbs

The barn owl's angle between the rachis and the barb on the outer vane was constant over the whole length of the feather and also, by and large, independent of the position of the feather on the wing. By contrast, the pigeon's angle changed depending on both parameters, except for feather p10. The acquired data on the pigeon is consistent with measurements by Ennos et al. [23]. The barb density in the barn owl decreased towards the tip of the feathers. A similar change was observed by Neuhaus et al. [11] in the tawny owl. In contrast to that, the pigeon's barb density increased only at the very end of the feather, again paralleling what Neuhaus et al. [11] measured in another non-specialist, the mallard duck.

The serration is a special characteristic of the barbs on the outer vanes of feathers p10 and gpc10 of the barn owl. Such serrations can also be found in other owls [11,25]. In some owls, like the African eagle-owl, the tawny owl [25] or long-eared owl the serrations also extend to p9 at those positions that form a secondary leading edge of the wing. Since the barn owl's feather p10 is very long and is the only feather to form the leading edge of the wing, we did not observe serrations on any other feather than p10 and gpc10. No serrations were found on the pigeon's wing.

It has often been claimed that serrations have an aerodynamic function [8-10,19,26]. However, quantitative data are rare. For the present report, the two-dimensional structure of the serrations was quantified. Sodermann [26,27] also presented data on serrations of the barn owl. His measurements yielded much longer serrations than those observed in this study, although he most likely studied the American subspecies *Tyto alba pratincola*, too. The differences in the results remain unclear. He tested several forms of serrations and observed that the noise produced by the wing was reduced by 4–8 dB for a large-scale rotor. By contrast, Schwindt and Allen [19] did not find such an effect. Neuhaus et al. [11] only found an effect during the take-off and landings phases, but not during ongoing flapping flight and gliding phases. The discrepancy in the results of the studies may arise from the different shapes of the investigated serrations and the different methods which were used.

The fringes at the edges of the feathers and the wings are a further specialisation of the owl's barbs. Fringes were observed on the pigeon's feathers only at their base, where they resemble the well-known plumulaceous barbs [16]. Graham [8], who mentioned fringes as a structure that would prevent the formation of noise-producing vortices, did only notice fringes to occur on the inner vane. Obviously, the situation is more complex, because fringes occurred on the outer vane as well as on the inner vane of all feathers studied. Nevertheless, it is believed that the fringes do have an aerodynamic and noise-reducing role, but further investigations are necessary to prove this.

### Morphology of barbules

Another noticeable difference between the pigeon and the barn owl was the difference in length of the pennula of the radiates. While the radiates were confined to the space between two barbs in the pigeon, the barn owl's pennula were so long that they were often overlapping the next three or four barbs. Similar observations were made in the African eagle owl by Taranto [28]. The long pennula were the source of the velvet-like surface of the barn owl's feathers. Graham [8] mentioned a noise-reducing function, which is plausible, but has not been proved yet. Of course, the elongated pennula increase the porosity of the feather surface. Porous materials are also discussed as noise absorbers [29], but the function of the velvet-like surface of the barn owl's feathers is still unclear. It is remarkable that the length of the pennula of the inner vane were increased towards the barb's tip in feather p10. Thus, the pennula of the covered areas were longer than those of the uncovered ones. In this area most of friction between feathers occurs while flapping the wings. Lentink et al. [30] showed a morphing effect of bird wings in different flying manoeuvres and flying speeds. Birds vary the angles at elbow and wrist to change the form and size of their wings. As a consequence, feathers rub against each other. As the barbs build up a parallel grooved structure by their shafts, this structure probably produces a high frequency noise while they rub against each other. A soft structure like the pennula is well suited to reduce these noises. Lilley [9] argued that the porosity of the owl's wing is unlikely to eliminate all sounds generated at high frequencies, since most of the sound emission has little to do with the wing's surface and would radiate away. The pennula may have a function in noise reduction beyond increasing porosity. It was observed that pennula length is increased in those areas where feathers overlap. Lilley [9] speculated that the noise may be eliminated at the location of its source. If the rubbing of the barb shafts is a source of noise, then the observations fit with Lilley's [9] speculation: elongated pennula should prevent noise generation or at least reduce it.

As mentioned before, differences in density of barbules and hooklets occurred between the two investigated species. Pigeons had more radiates per mm and more hooklets per hook radiate for the linkage of barbs than the barn owl. Additionally, the barn owl's barbs tapered towards their endings and the number of hooklets decreased to zero. The reduction of the number of hooklets is the basis for the formation of the fringes. Air transmissivity in the area of the fringes is very high. This feature might have an influence on the flow field and the boundary layers. Lilley [9] suggested that scatterings of the airflow are reduced or even eliminated by devices such as fringes.

## Conclusion

In this report we compare the morphology of barn owl flight feathers to pigeon flight feathers. We emphasise specific structures seen only in the barn owl. Barn owl feathers were in general larger than pigeon feathers indicating a lower wing load in this bird that would allow slow flight. The asymmetry of several parameters between the outer and inner vanes was more pronounced in the barn owl than the pigeon. This suggested a stiffer leading edge of the feathers due to the raches in addition to small outer vanes and a pliant and flexible inner vane. Both parameters influence the formation of a smooth wing surface during gliding flight or downstroke in flapping flight and help to minimise the flow resistance through the wing during upstroke in flapping flight. Owl feathers also had less radiates, longer pennula, and were more porous than pigeon feathers. This suggested that air could more easily pass from dorsal to ventral and vice versa in the owl feathers than in the pigeon feathers. The most conspicuous specialisations in the barn owl are the serrations at the leading edge of the wing, the fringes at the edges of each feather, and the velvet-like dorsal surface. The specialisations in the barn owl have been discussed in the context of the silent flight of the barn owl. However, convincing quantitative data are missing. The data presented here may serve as a basis for testing the influence of each specific feature on the owl's feathers on the air flow field and noise production.

## Methods

Wings of barn owls (*Tyto alba pratincola *Linnaeus) and pigeons (*Columba livia *Linnaeus) were prepared to carry out the morphological investigations. Barn owl wings were obtained from specimens of the institute's own colony that had been used in other experiments and were killed by perfusion under a permit of the local authorities (Landespräsidium für Natur, Umwelt und Verbraucherschutz Nordrhein Westfalen, Recklinghausen, Germany (LANUV)). Five wings of three different animals were prepared. Four Pigeons were received from a breeder and killed specifically for this study under a permit from LANUV. Thus, eight pigeon wings were obtained.

The morphological investigations included six remiges and six coverts for each species from different positions. For the barn owl, five feathers from position p10, and two, each from p9, p5, p1, s4, s8, gpc10, gpc9, gpc5, gpc1, gsc5 and gsc9 were acquired. Eight feathers from position p10 and two each from p9, p5, p1, s4, s8, gpc10, gpc9, gpc5, gpc1, gsc5 and gsc10 for the pigeon (Fig. 1A).

All feathers were removed from the wings and photographed with an 8-megapixel digital camera (Canon EOS 350D, Canon Inc., Tokio, Japan) with a 50 mm macro lens. Additionally, they were scanned by an Epson flat-bed scanner (Epson Perfection 3490 Photo, Seiko Epson Corporation, Tokio, Japan) with a resolution of 800 dpi from dorsal and ventral side.

Measurements took place at every 10% of the vane length. Parameters such as the depth of vane or the length of barbs were then normalised by the whole length of the vane for each single feather. The following parameters were extracted from the photos (Fig. 1B):

- Length of the rachis (whole length of the shaft including calamus).

- Depth d of the outer (ov) and inner vane (iv). From measurements between 10% and 90% of vane length (in remiges, resp. 20–80% in the coverts) an asymmetry index AI was derived, defined as:

AId=dov−divdov+div.
 MathType@MTEF@5@5@+=feaafiart1ev1aaatCvAUfKttLearuWrP9MDH5MBPbIqV92AaeXatLxBI9gBaebbnrfifHhDYfgasaacPC6xNi=xI8qiVKYPFjYdHaVhbbf9v8qqaqFr0xc9vqFj0dXdbba91qpepeI8k8fiI+fsY=rqGqVepae9pg0db9vqaiVgFr0xfr=xfr=xc9adbaqaaeGacaGaaiaabeqaaeqabiWaaaGcbaGaemyqaeKaemysaK0aaSbaaSqaaiabdsgaKbqabaGccqGH9aqpjuaGdaWcaaqaaiabdsgaKnaaBaaabaGaem4Ba8MaemODayhabeaacqGHsislcqWGKbazdaWgaaqaaiabdMgaPjabdAha2bqabaaabaGaemizaq2aaSbaaeaacqWGVbWBcqWG2bGDaeqaaiabgUcaRiabdsgaKnaaBaaabaGaemyAaKMaemODayhabeaaaaGaeiOla4caaa@4545@

AI may vary between -1 and 1 and is 0, if the inner and outer vanes have the same depth. It is positive (negative), if the outer vane is narrower (wider) than the inner vane.

- Length of barbs of the outer and inner vane. In this measurements the existence or lack of serrations and fringes on the outer as well as on the inner vane were investigated. If such structures were found their typical mean size and spacing was measured and indicated by a dotted line in the Figures 1B and 4. To reduce the influence of the plumulaceous barbs and abrasions at the feather tips, the mean size and spacing was calculated between 20–90% of the vane length for the remiges and between 20–80% for the coverts.

- Size of the outer and inner vane (the area was measured by counting the pixels and multiplying them by the size of one pixel). A similar asymmetry index as for the depth was calculated, defined as:

AIa=aov−aivaov+aiv.
 MathType@MTEF@5@5@+=feaafiart1ev1aaatCvAUfKttLearuWrP9MDH5MBPbIqV92AaeXatLxBI9gBaebbnrfifHhDYfgasaacPC6xNi=xI8qiVKYPFjYdHaVhbbf9v8qqaqFr0xc9vqFj0dXdbba91qpepeI8k8fiI+fsY=rqGqVepae9pg0db9vqaiVgFr0xfr=xfr=xc9adbaqaaeGacaGaaiaabeqaaeqabiWaaaGcbaGaemyqaeKaemysaK0aaSbaaSqaaiabdggaHbqabaGccqGH9aqpjuaGdaWcaaqaaiabdggaHnaaBaaabaGaem4Ba8MaemODayhabeaacqGHsislcqWGHbqydaWgaaqaaiabdMgaPjabdAha2bqabaaabaGaemyyae2aaSbaaeaacqWGVbWBcqWG2bGDaeqaaiabgUcaRiabdggaHnaaBaaabaGaemyAaKMaemODayhabeaaaaGaeiOla4caaa@4527@

- Angle between barbs and rachis on the inner and outer vane (the angle at the base of each barb was measured; the arms of the angle were placed in the middle of the rachis, resp. of the barb).

- Number of barbs (all barbs were counted and the density [n_b_/cm] was calculated).

The barbs of the feathers p10, s8 and gpc1 of both species were examined by using scanning electron microscopy in order to measure the fine structures. Barbs from the inner and outer vane were taken at four different positions of the vane (at 20%, 40%, 60% and 80%) (Fig. 1B). This results in eight barbs per feather that were analysed. Every barb was cut off at the rachis and placed with a glue pad on an aluminium specimen stub. Afterwards, the specimens were gold coated with a sputter coater (model: Hummer, Technics Inc., Alexandria, Virginia, USA, 10 mA, 1000 V, 7–9 min). Pictures were taken with a Cambridge Stereoscan 604 scanning electron microscope (Cambridge Instruments, Cambridge, UK) at 20%, 40%, 60% and 80% of the barb length. With respect to the much shorter barbs of the outer vane of feather p10, the positions of the pictures were changed to 25%, 50% and 75% of the barb length.

The following parameters were extracted (Fig. 1C):

- Number of hook and bow radiates per mm (n_hr_; n_br_).

- Angle (α) under which the barbules are attached to the barb (α_br_; α_hr_).

- Number of hooklets at the hook radiates (n_h_).

- Length of the barbules (base and pennulum).

The data was obtained from photographs using Adobe Photoshop CS (Adobe Systems, San Jose, California, USA) and ImageJ (National Institutes of Health, USA).

## Abbreviations

α_br _– angle between bow radiate and barb shaft

α_hr _– angle between hook radiate and barb shaft

a – area

AI_a _– asymmetry index of the area of the vanes

AI_d _– asymmetry index of the depth of the vanes

b – base

br – bow radiate

bs – barb shaft

d – depth

d_iv _– depth of inner vane

d_ov _– depth of outer vane

gpcX – greater primary covert No. X (see Fig. 1A)

gscX – greater secondary covert No. X (see Fig. 1A)

h – hooklet

hr – hook radiate

iv – inner vane

l_fr _– length of fringes

l_serr _– length of serration

n_b _– number of barbs (per cm)

n_br _– number of bow radiates (per mm)

n_h _– number of hooklets (per hook radiate)

n_hr _– number of hook radiates (per mm)

ov – outer vane

p – pennulum

pX – primary No. X (see Fig. 1A)

r -   radiate, radiate base

sX – secondary No. X (see Fig. 1A)

## Competing interests

The author(s) declare that they have no competing interests.

## Authors' contributions

TB carried out the morphometric data on the feathers and barbs and drafted the manuscript. TB and SK participated in the scanning electron microscopy studies and their analysis. MK participated in the photography analysis of the feathers. WB carried out the design of the study and participated in scanning electron microscopy. WS and HW designed and supervised the study, and coordinated the experiments. Both were also involved in drafting the manuscript. All authors read and approved the final manuscript
